# Supplementary material for: Cation and Anion Channelrhodopsins: Sequence Motifs and Taxonomic Distribution
Source: mBio. 2021 Jul 20;12(4):e01656-21. doi: 10.1128/mBio.01656-21 (PMC8406140; doi:10.1128/mBio.01656-21)
Supplement: TABLE S1 [file mbio.01656-21-st001.docx]

**Table S1.** Solution compositions in AxoPatch and SyncroPatch recordings.

Abbreviations: Asp, aspartate; EGTA, ethylene glycol tetraacetic acid; HEPES, 4-(2-hydroxyethyl)-1-piperazineethanesulfonic acid; LJP, liquid junction potential; NMDG, N-Methyl-D-glucamine. All concentrations are in mM.

|  | **NaCl** | **KCl** | **CsCl** | **CsF** | **CaCl_2_** | **MgCl_2_** | **EGTA** | **HEPES** | **NMDG*** | **Glucose** | **NaAsp** | **pH** | **LJP** |
| --- | --- | --- | --- | --- | --- | --- | --- | --- | --- | --- | --- | --- | --- |
| **Pipette standard** | — | 126 | — | — | 0.5 | 2 | 5 | 25 | — | — | — | 7.4 | — |
| **Pipette NaCl** | 126 | — | — | — | 0.5 | 2 | 5 | 25 | — | — | — | 7.4 | — |
| **Pipette CaCl_2_** | — | — | — | — | 68 | 2 | — | 25 | — | — | — | 4.4 | — |
| **Bath standard** | 150 | — | — | — | 1.8 | 1 | — | 10 | — | 5 | — | 7.4 | 1.1 |
| **Bath NaAsp** | — | — | — | — | 1.8 | 1 | — | 10 | — | 5 | 150 | 7.4 | -13 |
| **Bath pH 6.4** | 150 | — | — | — | 1.8 | 1 | — | 10 | — | 5 | — | 6.4 | 1.1 |
| **Bath NaCl 1.3** | 1.3 | — | — | — | 1.8 | 1 | — | 10 | 150 | 5 | — | 7.4 | 7 |
| **Bath KCl 1.3** | 1.3 | — | — | — | 1.8 | 1 | — | 10 | 150 | 5 | — | 7.4 | 10.2 |
| **Bath CaCl_2_ 1.8** | — | — | — | — | 1.8 | 1 | — | 10 | 150 | 5 | — | 7.4 | 1.6 |
| **Nanion Internal CsF** | 10 | — | 10 | 110 | — | — | 10 | 10 | — | — | — | 7.2 | — |
| **Nanion External Chip Fill** | 140 | 4 | — | — | — | — | — | 10 | — | 5 | — | 7.4 | — |
| **Nanion External Physiological** | 140 | 4 | — | — | 2 | 1 | — | 10 | — | 5 | — | 7.4 | — |
| **Nanion External NMDG 60** | 80 | 4 | — | — | 2 | 1 | — | 10 | 60 | 5 | — | 7.4 | — |

*NMDG stock (pH 9) was prepared from 1M NMDG and ~700 mM HCl.
