# Supplementary material for: Cation and Anion Channelrhodopsins: Sequence Motifs and Taxonomic Distribution
Source: mBio. 2021 Jul 20;12(4):e01656-21. doi: 10.1128/mBio.01656-21 (PMC8406140; doi:10.1128/mBio.01656-21)
Supplement: TABLE S2 [file mbio.01656-21-st002.docx]

**Table S2.** List of cryptophyte strains analyzed

Abbreviations: BEA, Banco Español de Algas, of the Universidad of Las Palmas de Gran Canaria, Spain; CCAC, Culture Collection of Algae at the University of Cologne, Germany; SCCAP, Scandinavian Culture Collection of Algae and Protozoa at the University of Copenhagen, Denmark; CCMP, Culture Collection of Marine Phytoplankton at the Provasoli-Guillard National Center for Marine Algae and Microbiota at Woods Hole Oceanographic Institution, USA; NIES, National Institute for Environmental Studies, Tsukuba, Japan.

|  | **Catalog number** | **Genus and species names** | **Habitat (growth medium)** | **ACRs found?** |
| --- | --- | --- | --- | --- |
|  | CCMP 0268 | *Chroomonas sp.* | Marine (ASP-12)* | No^#^ |
|  | CCAC 4037 B | *Chroomonas sp.* | Marine (ASP-12)* | No^#^ |
|  | NIES 1370 | *Chroomonas sp.* | Marine (ASP-12)* | No^#^ |
|  | CCAC 0173 B | *Chroomonas sp.* | Marine (ASP-H)* | No^#^ |
|  | CCAC 1627 B | *Chroomonas sp.* | Marine (ASP-H)* | Yes |
|  | CCAC 3782 B | *Chroomonas sp.* | Freshwater | No^#^ |
|  | CCAC 3670 B | *Chroomonas sp.* | Marine (ASP-H)* | No^#^ |
|  | BEA 0199B | *Cryptomonas cf. pyrenoidifera* | Freshwater | No^#^ |
|  | CCAC 0108 | *Cryptomonas gyropyrenoidosa* | Freshwater | No^#^ |
|  | CCAC 0031 | *Cryptomonas obovoidea* | Freshwater | No^#^ |
|  | CCAC 0064 | *Cryptomonas ovata* | Freshwater | No^#^ |
|  | SCCAP K0416 | *Geminigera sp.* | Marine (ASP-12)* | No^#^ |
|  | CCAC 1074 B | *Komma caudata* | Freshwater | No^#^ |
|  | CCMP 0760 | *Rhodomonas sp.* | Marine (ASP-12)* | Yes |
|  | CCAP 979/6 | *Rhodomonas sp.* | Marine (ASP-12)* | Yes |
|  | CCAC 1480 B | *Rhodomonas sp.* | Freshwater | Yes |
|  | CCAC 3787 B | *Rhodomonas sp.* | Marine (ASP-12)* | Yes |
|  | CCAC 3407 B | *Rhodomonas sp.* | Marine (ASP-12)* | Yes |
|  | CCAC 3799 B | *Rhodomonas sp.* | Marine (ASP-12)* | Yes |
|  | BEA 0603B | *Urgorri complanatus* | Marine (ASP-12)* | No^#^ |

*For media recipes see the CCAC website (https://www.uni-due.de/biology/ccac/).

^#^Our finding of no ACR transcripts in this species does not necessarily mean that they are not encoded by its genome and expressed; the transcript abundance may simply have been below the detection limit of our method.
